# Endoplasmic Reticulum Stress and Microvascular Endothelial Dysfunction in Diabetes

**DOI:** 10.4172/2155-6156.1000108e

**Published:** 2011-12-15

**Authors:** Modar Kassan, Maria Galán, Soo-Kyoung Choi, Khalid Matrougui

**Affiliations:** Department of Physiology, Hypertension and Renal Center of Excellence, Tulane University, 1430 Tulane Ave, New Orleans LA-70112, USA

Diabetes mellitus is a chronic metabolic disease characterized by hyperglycemia, due to deficiency in insulin or insulin resistance, and represents a major cause of morbidity and mortality in contemporary societies [1,2]. According to the world health organization (WHO), more than 285 million people worldwide suffered from diabetes of which 4 million died in 2010. The prevalence is expected to enhance to 380 million by 2030. Genetic and environmental factors associated with life style such as unhealthy diet, physical inactivity, harmful use of alcohol and tobacco, and obesity contribute to the increasing incidence of diabetes. The situation becomes extremely critical since type 1 and type 2 diabetes compromise the cardiovascular homeostasis. Based on the report of WHO and clinical studies, the direct cause of death for 80 % of diabetic patients is cardiovascular diseases.

Studies in human and experimental diabetic animal models have reported vascular dysfunction and structural arterial wall remodeling [3–5]. It is well known that endothelial dysfunction is an important risk factor of cardiovascular diseases [6,7]. Several hypothesis and mechanisms documented the relationship between diabetes and microvascular endothelial dysfunction [8,9], which include reduced endothelium-derived relaxing factor (EDRF) release and bioavailability, and enhanced endothelium-derived constricting factors release associated with augmented oxidative stress levels. Despite treatments have progressed, the development of novel effective treatments for patients with vascular complications in diabetes remains a major research goal.

The presence of insulin receptors on endothelial cells is well documented [10] but the role of insulin resistance at the level of the endothelial cell in vascular physiopathology is unclear. A number of studies in humans and genetically modified mice have demonstrated a close association between insulin resistance and nitric oxide (NO) bioactivity. Thus, Steinberg et al. [11] and Wheatcroft et al. [12] reported a direct link between insulin-induced vasodilatation and nitric oxide synthase (eNOS) activity. Additionally, Kuboki et al. [13] demonstrated that insulin regulates eNOS transcription in endothelial cells. Another study by Dr. Quon et al. [14] elucidated that insulin activates eNOS by insulin receptor tyrosine kinase (IRS-1) and phosphatidylinositol 3-kinase (PI3K)-dependent mechanisms. These studies support the concept that insulin resistance impairs vascular endothelium-dependent relaxation by PI3K-NO defect-dependent mechanisms. Additionally, It has been shown that insulin increases extracellular signal signal-regulated kinases 1/2 (ERK1/2) and endothelin converting enzyme activity leading to endothelin-1 release increase [15]. Insulin resistance and reduced insulin levels decreased PI3K-NO-dependent signaling, which trigger imbalance between NO and endothelin-1 responsible for impaired vascular endothelium-dependent relaxation [15]. Moreover, the mechanism by which insulin signaling becomes impaired in endothelial cells remains unclear. However, emerging evidence indicates that endoplasmic reticulum stress is an important factor in diabetes-induced pathology [16,17]. Therefore, endoplasmic reticulum stress could be a new potential intermediate signaling that explains the link between insulin resistance and vascular endothelial dysfunction. Various cellular stresses (ischemia, hypoxia, gene mutation, oxidative stress, and increased protein synthesis) lead to impairment of endoplasmic reticulum function, and create a state termed as endoplasmic reticulum stress that leads to the activation of a complex signaling network called the unfolded protein response (UPR) [18–21]. The UPR is regulated in the cell by three endoplasmic reticulum membrane-associated proteins that act as sensors of endoplasmic reticulum homeostasis. The three membrane bound proteins are protein kinase-like endoplasmic reticulum eukaryotic initiation factor 2α kinase (PERK), inositol requiring endoplasmic reticulum to nucleus signaling protein-1α (IRE1α) and activating transcription factor-6 (ATF6).

Metabolic and cardiovascular diseases such obesity, stroke, myocardial ischemia and diabetes are associated with endoplasmic reticulum stress [22–24]. Additionally, endoplasmic reticulum stress is considered as a key element in pancreatic beta cell dysfunction and peripheral insulin resistance in diabetes [25,26]. This topic is not discussed here, but a number of excellent reviews in this area have been published [27–29]. In this review, we will focus on endoplasmic reticulum stress as a new mechanism that plays an important role in vascular dysfunction in diabetes. Thus, Ozcan et al. [16,22] demonstrated the first link between insulin resistance and reticulum endoplasmic stress suggesting that ER stress disrupts the mechanism of insulin signaling in liver, adipose tissue and pancreas. Moreover, Ozcan’s group also showed that endoplasmic reticulum stress increases c-Jun N-terminal kinase (JNK) and catalytic IkappaB kinase subunits activity, and induces inflammation associated with IRS-1 signaling impairment [16,22] suggesting that endoplasmic reticulum stress is an important factor that probably links obesity, insulin resistance and diabetes to vascular endothelial dysfunction. These observations are supported by: 1) our recent publication indicating that treated type 2 diabetic mice with endoplasmic reticulum stress inhibitor reduced body weight and blood glucose and insulin levels [30], and 2) the occurrence of endoplasmic reticulum stress in endothelial cells in metabolic diseases [31–33], emphasizing that endoplasmic reticulum stress is a potential mechanism that contributes to the reduced nitric oxide release and bioavailability, which leads to vascular endothelial dysfunction. Importantly, it is more likely that endoplasmic reticulum stress impairs vascular function by inflammation and oxidative stress-dependent mechanisms. Thus, it has been shown that endoplasmic reticulum stress could facilitate eNOS uncoupling, which leads to increase in oxidative stress [34,35] and tumor necrosis factor-α (TNF-α) production. Furthermore, enhanced TNF-α level in endothelial cells [36] has also been reported to induce endoplasmic reticulum stress that inhibits IRS-1 signaling by JNK and nuclear factor kappa B-dependent mechanisms [37–39]. It is also important to mention that eNOS uncoupling, which leads to oxidative stress generation can also induce endoplasmic reticulum stress in endothelial cells [40]. Together, these studies suggest the existence of potential circle between eNOS uncoupling, oxidative stress, endoplasmic reticulum stress and inflammation responsible for vascular endothelial dysfunction. It is important for future studies to determine the intermediate signaling linking endoplasmic reticulum stress to eNOS uncoupling, oxidative stress, inflammation and vascular endothelial dysfunction.

Recently, we reported that epidermal growth factor receptor tyrosine kinase (EGFRtk) plays an important role in vascular endothelial dysfunction in type 2 diabetes. Thus, we demonstrated that increased EGFRtk phosphorylation contributes to resistance artery dysfunction in type 2 diabetes [41]. Additionally, previous studies showed that the inhibition of EGFRtk activity promotes vasodilatation and reduces elevated arterial blood pressure in spontaneous hypertensive rat (SHR) and in insulin resistance and hypertensive animal models [42–44]. We recently have demonstrated an increase in EGFRtk activity in type 1 diabetes that is responsible for vascular endothelial dysfunction (unpublished data). Importantly, the inhibition of EGFRtk not only improved vascular endothelial function in type 1 diabetic mice, but also reduced endoplasmic reticulum stress suggesting a relationship between endoplasmic reticulum stress and EGFRtk. Interestingly, the inhibition of endoplasmic reticulum stress also improves vascular endothelial function in type 1 diabetic mice (unpublished data). These results suggest that endoplasmic reticulum stress is down stream signaling to EGFRtk and is an important factor responsible for vascular endothelial dysfunction in type 1 diabetes.

All together, these studies provide evidence that endoplasmic reticulum stress is an important factor in vascular endothelial dysfunction and its inhibition should be considered as a therapeutic strategy to overcome diabetes-induced vascular pathology.
